# Artificial Intelligence for Individualized Radiological Dialogue: The Impact of RadioBot on Precision-Driven Medical Practices

**DOI:** 10.3390/jpm15080363

**Published:** 2025-08-08

**Authors:** Amato Infante, Alessandro Perna, Sabrina Chiloiro, Giammaria Marziali, Matia Martucci, Luigi Demarchis, Biagio Merlino, Luigi Natale, Simona Gaudino

**Affiliations:** 1 Advanced Radiology Center (ARC), Department of Radiology and Oncological Radiotherapy, Fondazione Policlinico Universitario Agostino Gemelli IRCCS, 00168 Rome, Italy; giammaria.marziali@policlinicogemelli.it (G.M.); biagio.merlino@policlinicogemelli.it (B.M.); luigi.natale@policlinicogemelli.it (L.N.);; 2 Facoltà di Medicina e Chirurgia, Università Cattolica del Sacro Cuore, 00168 Rome, Italy; alessandro.perna03@icatt.it (A.P.);; 3 UOC Endocrinology and Diabetology, Fondazione Policlinico Universitario A. Gemelli IRCCS, 00168 Roma, Italy

**Keywords:** artificial intelligence in radiology, conversational agents, natural language processing (NLP), clinical decision support systems, patient-centered communication

## Abstract

**Background/Objectives:** Radiology often presents communication challenges due to its technical complexity, particularly for patients, trainees, and non-specialist clinicians. This study aims to evaluate the effectiveness of RadioBot, an AI-powered chatbot developed on the Botpress platform, in enhancing radiological communication through natural language processing (NLP). **Methods:** RadioBot was designed to provide context-sensitive responses based on guidelines from the American College of Radiology (ACR) and the Radiological Society of North America (RSNA). It addresses queries related to imaging indications, contraindications, preparation, and post-procedural care. A structured evaluation was conducted with twelve participants—patients, residents, and radiologists—who assessed the chatbot using a standardized quality and satisfaction scale. **Results:** The chatbot received high satisfaction scores, particularly from patients (mean = 4.425) and residents (mean = 4.250), while radiologists provided more critical feedback (mean = 3.775). Users appreciated the system’s clarity, accessibility, and its role in reducing informational bottlenecks. The perceived usefulness of the chatbot inversely correlated with the user’s level of expertise, serving as an educational tool for novices and a time-saving reference for experts. **Conclusions:** RadioBot demonstrates strong potential in improving radiological communication and supporting clinical workflows, especially with patients where it plays an important role in personalized medicine by framing radiology data within each individual’s cognitive and emotional context, which improves understanding and reduces associated diagnostic anxiety. Despite limitations such as occasional contextual incoherence and limited multimodal capabilities, the system effectively disseminates radiological knowledge. Future developments should focus on enhancing personalization based on user specialization and exploring alternative platforms to optimize performance and user experience.

## 1. Introduction

Radiology plays a critical role in modern medicine, offering essential imaging services that support accurate diagnosis and effective treatment planning [1,2,3]. However, navigating the complexities of radiology, such as understanding exam indications, patient preparation instructions, and contraindications, can be challenging for patients, medical trainees, and non-radiologist clinicians [4]. These challenges often lead to miscommunication, unnecessary imaging, or delays in care. To address these issues, we developed a healthcare chatbot service, hereafter referred to as the AI-guide bot, designed to provide accurate, timely, and accessible information about radiology exams. The chatbot leverages advanced natural language processing (NLP) to simulate human-like conversations and deliver reliable guidance based on established medical guidelines. Beyond its informational value, the AI-guide bot offers significant utility and cost-effectiveness compared to traditional human-based support systems. Human resources such as radiologists, nurses, and administrative staff are often limited and costly, especially in high-demand or resource-constrained environments. By automating responses to frequently asked questions and standardizing the delivery of information, the chatbot reduces the burden on healthcare professionals, allowing them to focus on more complex or urgent tasks. Moreover, the chatbot operates 24/7, ensuring continuous availability without the need for shift scheduling or overtime compensation. This round-the-clock accessibility enhances patient engagement and satisfaction, particularly for individuals seeking information outside regular clinic hours. From a cost perspective, the initial investment in developing and deploying the chatbot is offset by long-term savings in labor, training, and operational efficiency. Additionally, the bot minimizes the risk of human error in conveying standardized information, thereby improving the overall quality of care.

In summary, the integration of an AI-powered chatbot into radiology services not only addresses informational gaps but also represents a scalable, cost-effective solution that aligns with the goals of modern, patient-centered healthcare.

## 2. Background and Objective

Radiology is a cornerstone of diagnostic medicine, yet its complexity often creates barriers for those outside the specialty. Patients frequently struggle to understand the purpose and preparation requirements for imaging exams, while medical trainees and non-radiologist clinicians may lack the specialized knowledge needed to select appropriate imaging modalities or interpret procedural protocols. Even among radiologists, sub specialization can lead to knowledge gaps in areas outside their immediate expertise [5].

In this context, the need for a reliable, accessible, and scalable source of radiology-related information becomes evident. Traditional methods—such as printed guidelines, static web pages, or direct consultation with radiologists—are often insufficient due to their limited interactivity, time constraints, and variability in availability.

The objective of this study is to develop and evaluate a conversational AI system, referred to as the AI-guide bot, that leverages advanced natural language processing (NLP) to bridge these informational gaps. The chatbot is designed to serve as a dynamic, on-demand resource capable of providing accurate and up-to-date information on radiology exam indications, contraindications, preparation steps, and post-procedure care. It also permits interacting with patients in a user-friendly manner to clarify doubts, reduce anxiety, and improve compliance with exam protocols. In reducing the complexity of unfamiliar radiological language, RadioBot allows patients to be more engaged in their healthcare journey. The goal of individualized medicine is to promote autonomy and informed consent, supporting medical trainees and residents by offering quick, context-aware explanations that reinforce learning and clinical decision-making; assisting non-radiologist clinicians (such as general practitioners, emergency physicians, and surgeons) by guiding appropriate imaging choices and helping avoid unnecessary or redundant exams; and enhancing collaboration among radiologists, especially in multidisciplinary settings, by offering standardized references and reminders for less familiar procedures [6].

By integrating authoritative guidelines from different Radiological Society and sources such as the American College of Radiology (ACR) and the Radiological Society of North America (RSNA), the AI-guide bot ensures that its responses are not only conversational but also clinically sound. Its ability to operate in multiple languages further broadens its accessibility and relevance in diverse healthcare environments.

Other than its immediate use in clinical settings, the AI-guide bot represents a general shift towards embracing intelligent systems in healthcare education and communication. As radiology continues to evolve with increasingly advanced imaging modalities and protocols, there is the demand for context-sensitive, real-time supportive tools more acutely. The AI-advice bot addresses this need in turn by being not merely an information repository, but an interactive aide with the capacity to modulate responses based on user profiles, clinical contexts, and language choices. This mouldability is particularly beneficial in high-pressure environments such as emergency departments, where rapid decision-making is critical and access to radiology expertise may be at risk.

Secondly, the chat interface of the bot lowers the threshold of engagement, allowing for quicker access to and understanding of radiological information by users with varying levels of medical literacy. This is especially important in multilingual and multicultural healthcare organizations, where communications obstacles can compromise patient safety and quality care. By applying clear language explanations and dual language capacity, the AI-guide bot enhances the inclusivity and equality of healthcare delivery [7].

Educationally, the bot may serve as an adjunct learning tool for medical students and residents with real-time explanation of concepts about imaging as well as reinforcement of evidence-based practice. Its integration within EHR systems or clinical decision support systems could also further standardize workflows, reduce diagnostic errors, and support interdisciplinary collaboration. Lastly, the AI-guide bot demonstrates how artificial intelligence might be employed not to replace human know-how, but to augment it—increasing radiological expertise for broader dissemination, more convenient actionability, and more alignment with the principles of new, patient-focused medicine.

The chatbot aims to democratize access to radiology knowledge, reduce the cognitive load on healthcare professionals, and empower patients with the information they need to participate actively in their care.

## 3. Materials and Methods

To develop and evaluate the AI-guide bot, we employed a multi-step methodology combining knowledge base construction, chatbot configuration, and user-centered testing. The chatbot was implemented using Botpress (version 12.32.0, Botpress Inc., Quebec City, QC, Canada), a free conversational AI platform that supports integration of structured knowledge and natural language understanding. The knowledge base was populated using a curated collection of authoritative PDF documents, including clinical guidelines and procedural instructions from the American College of Radiology (ACR, Reston, VA, USA), the Radiological Society of North America (RSNA, Oak Brook, IL, USA), and other professional sources (Appendix A). These documents were uploaded into the Botpress platform under a dedicated knowledge tab, where they were automatically indexed to support semantic search and retrieval.

The chatbot was configured to respond to user queries in natural language, leveraging the indexed content to generate contextually appropriate and medically accurate responses. The system was designed to handle a wide range of radiology-related topics, including exam indications, patient preparation, contraindications, procedural details, and post-exam care. The interface was tested only in English ensure maximum compatibility and understanding by the bot. The chatbot fuses standardized guidelines with natural language processing to provide a personalized approach to communication that reflects patient-centered care, enabling each user to receive contextually appropriate and clinically relevant information.

Botpress integrates with external providers such as OpenAI (San Francisco, CA, USA), Anthropic (San Francisco, CA, USA), Groq (Mountain View, CA, USA), Hugging Face (Paris, France and New York, USA), and more. The LLMs used depend on the API you connect or configure within the platform. Specifically, it is connected to GPT-4o (version released May 2024), released in May 2024. It is a unified multimodal model that supports text, images, and audio, with a context window of 128,000 tokens. The model is designed for natural and fluid interactions, perfect for custom bots that require real-time responses, multilingual support, and the ability to process visual or audio content.

To assess the chatbot’s performance and usability, we conducted a structured evaluation involving 12 participants representing six distinct user profiles: 4 patients without medical training, 4 residents (two radiology medical residents and two general clinician residents), and 4 specialists (two radiologists). Each participant was instructed to interact with the chatbot by posing a series of questions relevant to their clinical or informational needs. The chatbot’s responses were collected and subsequently evaluated using a standardized satisfaction and quality assessment scale (Table 1), also derived from MOS-AI Score (doi: 10.21037/jmai-24-153).

During the evaluation phase, each participant posed a series of questions aligned with their typical informational needs or clinical responsibilities. The chatbot responded in real time, drawing from the structured knowledge base to deliver concise and medically accurate answers. The responses were subsequently reviewed and rated by the participants using a standardized quality and satisfaction scale.

Evaluation criteria included comprehensibility, accuracy, and perceived usefulness of the responses. This approach allowed for a comprehensive assessment of the chatbot’s effectiveness across a heterogeneous range of users, reflecting real-world scenarios in which such a tool might be deployed. No statistical analysis was used; the study was designed as an exploratory investigation.

## 4. Results

The AI-guide bot successfully demonstrated its ability to engage in natural language conversations and provide accurate, contextually relevant information regarding a wide range of radiology exams. The chatbot could respond to queries covering modalities such as magnetic resonance imaging (MRI), computed tomography (CT), X-rays (XR), and ultrasound (US). Responses were generated based on the indexed content from the uploaded guidelines and were tailored to the specific phrasing and intent of each user query.

Across all user groups, the chatbot received high ratings for clarity, relevance, and accuracy. Patients appreciated the simplicity and accessibility of the language used, which helped reduce anxiety and improve understanding of exam procedures. Medical trainees found the bot particularly useful for reinforcing their knowledge and clarifying procedural details, while clinicians valued the quick access to standardized information that could support decision-making in time-sensitive contexts. Radiologists and other specialists, although already familiar with the content, acknowledged the bot’s potential as a support tool for interdisciplinary communication and patient education.

**Figure 1:**
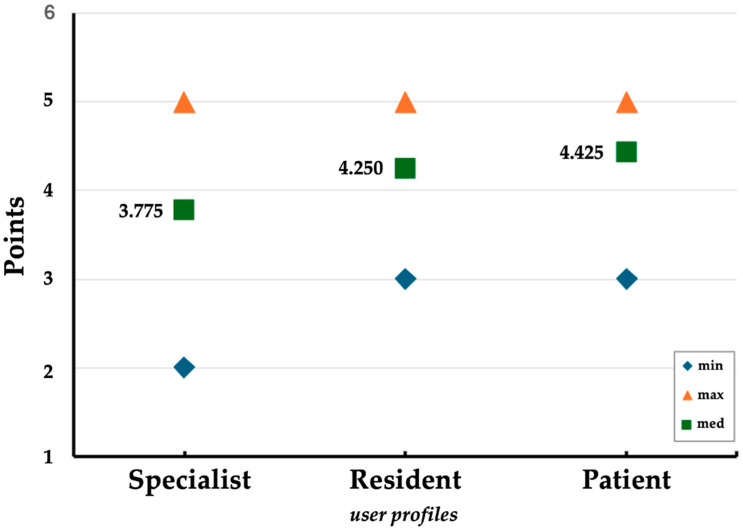
Comparison of minimum, median, and maximum rating scores for specialists, residents, and patients. Each group shows consistent maximum scores (5), while specialists have the lowest minimum rating (2). Median ratings increase progressively from specialists (3.775), to residents (4.25), to patients (4.425), indicating a trend toward higher median ratings from more general to more specialized care recipients.

The analysis revealed distinct patterns across the specialist, resident, and patient groups. The mean scores were highest among patients (mean = 4.425), followed by residents (mean = 4.250), and lowest among specialists (mean = 3.775). While the minimum and maximum scores for both residents and patients ranged from 3 to 5, specialists exhibited a broader range, with scores spanning from 2 to 5 (Figure 1). This suggests a higher degree of variability in the evaluations provided by specialists.

**Figure 2:**
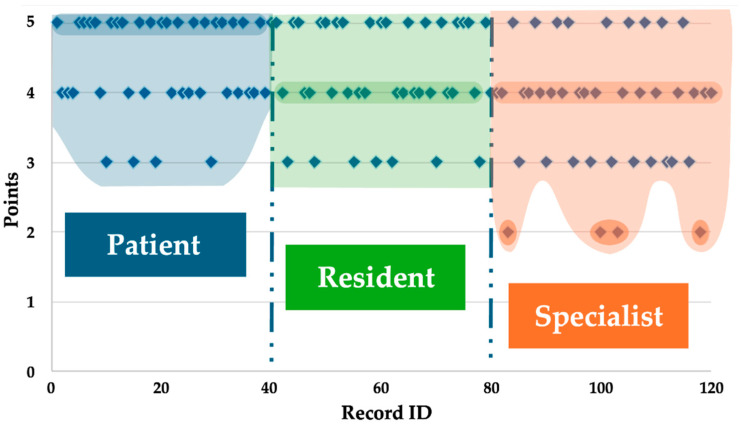
Distribution of ratings (1–5 scale) across three groups—patients, residents, and specialists—based on performance scores. The x-axis represents individual cases or assessments, while the y-axis shows the rating level. Each colored region (blue for patients, green for residents, and orange for specialists) highlights the density and variability in ratings within each group.

The distribution of maximum scores (i.e., score = 5) further supports this trend; patients assigned the highest number of top scores (n = 21), followed by residents (n = 17) and specialists (n = 9). The frequency of score 4 was relatively consistent across groups—patients (n = 15), specialists (n = 17), and residents (n = 16). However, score 3 was more frequently observed among specialists (n = 10) compared to residents (n = 7) and patients (n = 4), indicating a more critical or reserved evaluation pattern among specialists. Notably, score 2 was exclusively reported by specialists (n = 4), further highlighting their distinct response profile (Figure 2).

Overall, the results from residents and patients were largely overlapping, with patients showing a slightly higher average score (Table 2). In contrast, specialists demonstrated a more conservative evaluation pattern, both in terms of lower average scores and a wider distribution, including the lowest observed ratings.

## 5. Discussion

The integration of AI-powered chatbots into clinical practice has been explored across several medical specialties, including ophthalmology, urology, and endoscopy (Table 3).

In ophthalmology, for instance, chatbots have been evaluated for their ability to answer fact-based questions, assist in clinical decision-making, and support research activities. Similar applications have been reported in urology and gastroenterology, where conversational agents have been used to guide patients through procedural preparations and post-operative care, demonstrating improvements in patient engagement and adherence to clinical protocols.

While these studies highlight the growing utility of chatbots in healthcare, most have focused on either patient-facing applications or the accuracy of responses to standardized clinical queries. In contrast, our study introduces a novel dimension by evaluating the chatbot’s performance across a spectrum of user profiles with varying levels of medical expertise. This includes patients, medical trainees, non-radiologist clinicians, surgeons, and radiologists. This comparative approach allowed us to assess not only the informational accuracy of the chatbot but also its perceived usefulness and adaptability to different cognitive and professional contexts.

A key finding from our evaluation is the inverse correlation between users’ domain-specific expertise and their perceived efficiency of the chatbot’s responses. Participants with limited radiological training, such as patients and non-radiology residents, reported greater informational value from the chatbot, as it provided structured, accessible explanations that addressed their knowledge gaps. RadioBot leverages personalized medical education for both residents and junior clinicians by providing cognitive explanations that are tailored to their stage of development, helping them to acquire knowledge progressively forming forms of personalized learning.

Conversely, users with advanced expertise, such as radiologists and experienced clinicians, often found the responses to be less informative [10], as the chatbot primarily reiterated information they already possessed [8]. However, even among expert users, the chatbot was appreciated as a time-saving tool that facilitated rapid access to standardized information, reducing the need for manual searches through guidelines or institutional protocols.

These findings suggest that while AI chatbots are highly effective in disseminating structured medical knowledge, their utility is modulated by the user’s baseline familiarity with the subject matter. For novice users, the chatbot functions as an educational and navigational aid. For experts, it serves more as a tool for workflow optimization and information retrieval. This dual utility underscores the importance of tailoring chatbot design and content delivery to accommodate varying user needs and expectations [9,11,12].

Among the study’s limitations, it is important to note that the evaluation was conducted using Botpress, a partially free conversational AI platform, under the constraints of its free-tier plan. Consequently, the system was subject to monthly usage quotas, which may have affected the continuity and depth of user interactions. A notable limitation observed was the occasional loss of contextual coherence during extended conversations, resulting in fragmented or inconsistent responses (screenshots...). Additionally, in cases where the input was not clearly understood, the system tended to repeat generic replies rather than seeking clarification or adapting its response strategy (screenshots...). Despite being prompted, Botpress did not generate images, indicating a limitation in multimodal capabilities within the tested configuration.

Botpress was selected primarily for its accessibility and ease of configuration, which made it a practical choice for initial experimentation. However, to obtain a more comprehensive evaluation of conversational AI performance, future studies should consider testing alternative chatbot platforms and other LLMs to enable broader comparisons in terms of functionality, contextual understanding, and user experience.

Other limitations linked to the methodology could certainly be improved in future, expanded, and further randomized. Increasing the number of participants and the volume of interactions would also enhance the robustness and generalizability of the findings.

The findings of the research not only substantiate the growing use of AI-powered chatbots in clinical environments but also open new avenues for their strategic application in diverse healthcare settings. While current applications in ophthalmology, urology, and gastroenterology have already established the potential of the chatbot in supporting patient education and procedural compliance, our research introduces a more advanced understanding of chatbot utility in embracing a multi-profile evaluation practice. This strategy recognizes that the utility of conversational agents is not consistent but is dependent on the user’s background, expectations, and cognitive requirements. By measuring RadioBot’s performance over a range of users (from laypeople to radiologists) we were able to discern unique patterns in perceived usefulness, which can be used to guide future design and deployment efforts.

Perhaps the most perceptive observation is the way in which the chatbot supports gaps in training for junior clinicians and medical trainees. These tend to be the users operating under high-stakes situations with limited access to live mentorship or reference texts. RadioBot’s ability to give contextual, structured explanations in real-time enables the application of clinical reasoning and reinforces learning through repetition and clarity. This form of tailored medical education continues with the contemporary pedagogical frameworks that emphasize adaptive learning and competency-based advancement. In addition, the dialog-based format of the chatbot encourages active as opposed to passive engagement, which has been shown to enhance retention and comprehension over passive reading of fixed guidelines.

For the professional users, the utility of the chatbot lies not in learning but in efficiency in workflow. Radiologists and experienced clinicians, who are already familiar with procedural algorithms, value the chatbot’s quick access to pre-approved data, saving time searching within institutional databases or consulting paper manuals. This time saving is particularly precious in multidisciplinary settings, where prompt access to imaging guidelines may enhance collaborative decision-making and reduce delays in patient management. The possible integration of the chatbot into clinical decision support tools or electronic health record (EHR) systems can further augment this benefit to become a standard component of clinical workflow.

Other than these benefits, there are some technical and functional constraints that are highlighted in the study that have to be addressed for enhancing chatbot performance. The use of Botpress, while helpful from a positioning and ease of installation perspective, imposed restrictions on contextual flow and volume of interaction. Extended dialogs often provided fragmented responses, and the system’s lack of ability to clarify imprecise inputs or produce visual aids restricted its utility in certain situations. Such issues make the importance of selecting robust AI platforms and large language models (LLMs) with multimodal communication, contextual memory, and dynamic response strategies clear. Future research has to explore other platforms with more abilities, such as GPT-based platforms or rule-based logic with deep learning hybrid models, for maximizing conversational depth and responsiveness.

In short, the study aims to test RadioBot and its potential as a versatile tool in clinical education, patient empowerment, and workflow efficiency. Its success, however, depends on user profiling, platform functionality, and integration strategies. As healthcare professionals increasingly embrace digital transformation, the deliberate design and implementation of conversational agents will be paramount in making these technologies live up to their promise to improve care quality, accessibility, and efficiency.

## 6. Conclusions

The integration of artificial intelligence (AI) into clinical pathways is no longer a figment of the imagination but an inevitable reality, with AI-powered chatbots being a viable option to enable improved communication, education, and decision-making in medicine. In summary, our study contributes to the growing body of evidence supporting the use of AI chatbots in clinical settings, while also highlighting the importance of user profiling in evaluating and optimizing chatbot performance.

This study demonstrates the feasibility and value of deploying a radiology-focused AI chatbot to support diverse user groups in accessing standardized, guideline-based information.

This study contributes to such a nascent framework by validating the feasibility, usefulness, and adaptability of an AI-powered chatbot, RadioBot, designed for radiology, to enable informational closure across a spectrum of individuals from patients to veteran clinicians. Through the use of natural language processing (NLP) and clinical guidelines based on authoritative sources, RadioBot offers a scalable, interactive solution to the age-old challenge of presenting complex radiological information in an actionable and context-sensitive format.

One of the most significant contributions of this study is the emphasis on user profiling as a determinative factor in the evaluation of chatbots. In contrast to previous implementations, which were narrowly focused on either patient education or clinician support, our approach recognized the heterogeneity of end-users and sought to evaluate the performance of the chatbot under varying degrees of medical expertise. This multifaceted evaluation plan revealed a rich picture of how AI tools can be configured to meet the particular requirements of various types of users. Inexperienced clinicians, such as trainees and patients, benefited the most regarding informational gain and confidence to understand radiological procedures. Seasoned users—radiologists and experienced clinicians—appreciated the chatbot’s ability to provide access to complex, guideline-based information, with time-saving and reduced cognitive load.

RadioBot’s deployment illustrates the movement towards individualized healthcare, where digital technologies are purposefully displayed to ensure that medical communications address the information and emotional needs of continuously changing patient populations.

This double use is testimony to the flexibility of AI chatbots in a clinical environment. For the patient, RadioBot is an electronic pal that myth-busts concerning radiological exams, clarifies steps to prepare, and defuses fear by offering reassuring, concise messages. That supports overall patient-centered care goals, where informed consent, autonomy, and reassurance are paramount. For radiology non-specialist clinicians, the chatbot acts as a just-in-time learning resource, enabling accurate imaging choices, avoiding inappropriate examinations, and increasing adherence to best practice. Within multi-disciplinary settings, where multi-specialty coordination is required, RadioBot may become a point of reference that can ensure consistency and restrict variability of radiological decision-making.

Built using Botpress and populated with authoritative clinical content, the chatbot effectively addressed questions related to radiological procedures.

The development of the bot on the open-source conversational AI platform Botpress optimizes transparency, customizability, and scalability in healthcare AI technology. Packing the bot with content from trusted sources such as the American College of Radiology (ACR) and the Radiological Society of North America (RSNA) ensured that not only was the bot conversationally fluent but also that its answers were clinically accurate. This coupling of evidence-based material with sophisticated NLP functionality sets a precedent for how future AI-powered tools will achieve balance between ease of access and precision.

Yet the research also uncovered a number of limitations and challenges that should be explored further. Firstly, while the chatbot performed well in test simulation settings, bringing it into actual clinical practice may be disrupted by confrontation with problems such as interoperability with electronic health records (EHRs), concerns about data protection, and resistance from users not used to conversational interfaces. Secondly, while the current version of RadioBot is multilingual, it will still struggle with comprehending complex medical terminology or cultural disparities in health communication. To overcome these limitations, there will be a continuous improvement of the NLP models, knowledge base extension, and feedback from users iteratively.

Unlike prior implementations in specialties such as ophthalmology, urology, and endoscopy, our approach uniquely evaluated the chatbot across a spectrum of users with varying levels of medical expertise. Results revealed that while less experienced users benefited most in terms of informational gain, even expert users found the tool valuable for streamlining access to detailed content and saving time.

Another direction for future research is the deployment of adaptive response mechanisms. While our research categorized users manually based on expertise levels, subsequent versions of the chatbot can be integrated with real-time user profiling to adjust the depth, tone, and complexity of responses dynamically. For instance, a student of medicine inquiring regarding MRI contraindications would be provided with a detailed, pathophysiology-oriented explanation, while a patient would be presented with a simplified, reassurance-focused explanation. Individualization would not only raise the level of user satisfaction, but it would also strengthen the educational and clinical value of the chatbot.

Furthermore, integration with clinical decision support systems (CDSS) opens up new avenues for the use of RadioBot. Integration with EHR systems would enable clinicians to receive context-dependent recommendations during patient consultations, e.g., proposed imaging modalities based on patient symptoms and history. This would turn the chatbot into a dynamic component of clinical reasoning, not a passive provider of information, which would further amplify its impact on care quality and efficiency.

From a broader perspective, RadioBot’s success signals a growing recognition of the revolutionary role that AI plays in healthcare communication. As medical knowledge marches forward at historically unprecedented velocities, traditional forms of information provision—textbooks, static guidelines, even peer consultations—are becoming progressively scarce. AI chatbots offer an out-of-the-box solution that can keep pace with shifting evidence, learn about user needs, and act at scale. They also democratize access to expert information, countering inequalities in the provision of healthcare on geographic, language, and socio-economic grounds.

These findings underscore the dual role of AI chatbots: as educational aids for novices and as efficient tools for experienced professionals. Future developments should consider adaptive response mechanisms that adjust the depth and complexity of information based on the user’s expertise, thereby enhancing the chatbot’s relevance and effectiveness across diverse clinical roles.

It is worth noting, however, that the ethical implications of applying AI in clinical settings should not be overlooked. Ensuring transparency, accountability, and user trust is essential, particularly where chatbots are used to convey medical information capable of affecting patient choice or clinical practice. In our work, we addressed this by clearly labeling the chatbot as an information tool, not a diagnostic one, and by grounding its responses in verifiable clinical advice. Future work can continue to explore frameworks for the ethical use of AI, including methods for auditing chatbot operation, reducing misinformation, and safeguarding user data [13].

In general, the research validates the potential for AI chatbots like RadioBot to enable radiological communication, education, and decision-making across diverse user groups. Through the collaboration of advanced NLP, expert content, and human-centered design, RadioBot illustrates how digital technology can drive the goals of modern healthcare: enabling access, affirming comprehension, and improving collaboration. Looking ahead, continued innovation in these kinds of tools—guided by systematic evaluation, ethical standards, and user input—will be key to realizing the potential of AI in healthcare. The journey toward intelligent, fair, and responsive health systems is already far along, and conversational AI is a leading driver of that journey [14].

## Figures and Tables

**Table 1:** The quality evaluation scale (0–5 point). Based on mean opinion score (https://en.wikipedia.org/wiki/Mean_opinion_score accessed on 15 April 2025), revised and also based on MOS-AI score from 10.21037/jmai-24-153.

Point	Description
0	No understanding of the question/total misunderstanding or answer completely irrelevant to the question as a null answer
1	Poor understanding of the question, missing or incorrect information and too general description lacking essential information
2	Poor understanding, the answer and information may be related to the question but only a little valuable information is present
3	Correct understanding of the question and satisfactory answer, sufficient content, missing details or inaccurate description, but correct answer
4	Correct understanding associated with a description only partially incomplete or imprecise of the details
5	Perfect understanding, even of the details, combined with a precise and timely answer

**Table 2:** Absolute values sampled from each category, based on the assigned score.

	Specialist	Resident	Patient
**5-point**	9	17	21
**4-point**	17	16	15
**3-point**	10	7	4
**2-point**	4	0	0

**Table 3:** Comparative table with previous research.

Comparative Table with Previous Research
▪ Performance of large language models on benign prostatic hyperplasia frequently asked questions. Prostate. 1 April 2024 doi: 10.1002/pros.24699 [1]
▪ Exploring the potential of Chat-GPT as a supportive tool for sialendoscopy clinical decision making and patient information support. Eur Arch Otorhinolaryngol. 2024 Apr;281 (4):2081–2086. doi: 10.1007/s00405-023-08104-8 [3]
▪ Accuracy of Information given by ChatGPT for patients with Inflammatory Bowel Disease in relation to ECCO Guidelines. J Crohns Colitis. 23 March 2024:jjae040. doi: 10.1093/ecco- jcc/jjae040 [4]
▪ ChatGPT as an information tool in rhinology. Can we trust each other today? Eur Arch Oto-rhinolaryngol. 4 March 2024. doi: 10.1007/s00405-024-08581-5 [5]
▪ Enhancing Patient Communication With Chat-GPT in Radiology: Evaluating the Efficacy and Readability of Answers to Common Imaging-Related Questions. J Am Coll Radiol. 2024 Feb;21 (2):353–359. doi: 10.1016/j.jacr.2023.09.011 [7]
▪ Accuracy and Completeness of ChatGPT-Generated Information on Interceptive Orthodontics: A Multicenter Collaborative Study. J Clin Med. 27 January 2024;13 (3):735. doi: 10.3390/jcm13030735 [8]
▪ Urological Cancers and ChatGPT: Assessing the Quality of Information and Possible Risks for Patients. Clin Genitourin Cancer. 2024 Apr;22 (2):454–457.e4. doi: 10.1016/j.clgc.2023.12.017 [9]

## Data Availability

No publicly available datasets were used or generated during the study. The data supporting the findings of this study are not publicly available due to privacy or ethical restrictions.

## Appendix A. The Table Shows the File Names and the Source from Which They Were Taken (Free Download)

**Source**	**PDF File**
https://radiopaedia.org (Accessed on 15 April 2025)	CT head summary
https://geiselmed.dartmouth.edu/ (Accessed on 15 April 2025)	Guidelines For Use Of Oral Contrast In Abdominal Imaging Ct Exams
SIRM	SIRM protocol
**MRI**	
https://radiopaedia.org (Accessed on 15 April 2025)	Abdominal pain in pregnancy protocol
https://radiopaedia.org (Accessed on 15 April 2025)	Adrenal glands protocol
https://radiopaedia.org (Accessed on 15 April 2025)	Anal canal cancer protocol
https://radiopaedia.org (Accessed on 15 April 2025)	Endometrial carcinoma protocol
https://radiopaedia.org (Accessed on 15 April 2025)	Liver protocol
https://radiopaedia.org (Accessed on 15 April 2025)	Magnetic resonance cholangiopancreatography
https://radiopaedia.org (Accessed on 15 April 2025)	MR defecating proctography
https://radiopaedia.org (Accessed on 15 April 2025)	MR elastography
https://radiopaedia.org (Accessed on 15 April 2025)	MR enteroclysis
https://radiopaedia.org (Accessed on 15 April 2025)	MR enterography
https://radiopaedia.org (Accessed on 15 April 2025)	Pelvic cervical carcinoma protocol
https://radiopaedia.org (Accessed on 15 April 2025)	Pelvic protocol for endometriosis
https://radiopaedia.org (Accessed on 15 April 2025)	Perianal fistula protocol
https://radiopaedia.org (Accessed on 15 April 2025)	Prostate MRI protocol
https://radiopaedia.org (Accessed on 15 April 2025)	Rectal cancer protocol
State-of-the-art pancreatic MRI. doi: 10.2214/ajr.10.4421. [15]	State-of-the-Art Pancreatic MRI
**Breast**	
https://www.hopkinsmedicine.org (Accessed on 15 April 2025)	Breast Magnetic Resonance Imaging preparation
https://radiopaedia.org (Accessed on 15 April 2025)	Breast MRI
https://www.dartmouth-health.org (Accessed on 15 April 2025)	Breast-Protocols mri
**Chest/Heart**	
https://radiopaedia.org (Accessed on 15 April 2025)	ARVC protocol
https://radiopaedia.org (Accessed on 15 April 2025)	Cardiac gating
https://radiopaedia.org (Accessed on 15 April 2025)	Cardiac iron overload protocol
https://radiopaedia.org (Accessed on 15 April 2025)	Cardiac ischemia protocol
https://radiopaedia.org (Accessed on 15 April 2025)	Hypertrophic cardiomyopathy protocol
https://radiopaedia.org (Accessed on 15 April 2025)	Myocardial viability protocol
https://radiopaedia.org (Accessed on 15 April 2025)	Myocarditis protocol
https://radiopaedia.org (Accessed on 15 April 2025)	T1 mapping
https://radiopaedia.org (Accessed on 15 April 2025)	T2 mapping
https://radiopaedia.org (Accessed on 15 April 2025)	T2star mapping myocardium
https://radiopaedia.org (Accessed on 15 April 2025)	Thymic MRI
**Musculoskeletal**	
https://radiopaedia.org (Accessed on 15 April 2025)	Ankle protocol
https://radiopaedia.org (Accessed on 15 April 2025)	Anterior chest wall protocol
https://radiopaedia.org (Accessed on 15 April 2025)	Finger protocol
https://radiopaedia.org (Accessed on 15 April 2025)	Hip protocol
https://radiopaedia.org (Accessed on 15 April 2025)	Knee protocol
https://radiopaedia.org (Accessed on 15 April 2025)	Mid- and forefoot protocol (MRI)
https://radiopaedia.org (Accessed on 15 April 2025)	MRI elbow protocol
https://radiopaedia.org (Accessed on 15 April 2025)	MSK pelvis protocol
https://radiopaedia.org (Accessed on 15 April 2025)	sacroiliac joint protocol
https://radiopaedia.org (Accessed on 15 April 2025)	Shoulder protocol
https://radiopaedia.org (Accessed on 15 April 2025)	Wrist protocol
**Neuroradiology**	
https://radiopaedia.org (Accessed on 15 April 2025)	Brain infection protocol
https://radiopaedia.org (Accessed on 15 April 2025)	Brain screen protocol
https://radiopaedia.org (Accessed on 15 April 2025)	Brain stereotaxis protocol
https://radiopaedia.org (Accessed on 15 April 2025)	Brain trauma protocol
https://radiopaedia.org (Accessed on 15 April 2025)	Brain tumor protocol
https://radiopaedia.org (Accessed on 15 April 2025)	Demyelination protocol
https://radiopaedia.org (Accessed on 15 April 2025)	Diffusion tensor imaging and fiber tractography
https://radiopaedia.org (Accessed on 15 April 2025)	Epilepsy protocol
https://radiopaedia.org (Accessed on 15 April 2025)	MR Brain fMRI Neuro Protocol
https://radiopaedia.org (Accessed on 15 April 2025)	MRI BRAIN SUMMARY
https://radiopaedia.org (Accessed on 15 April 2025)	MRI protocol for neurodegenerative diseases assessment
https://radiopaedia.org (Accessed on 15 April 2025)	MRI Sequences in Head & Neck Radiology—State of the Art
https://radiopaedia.org (Accessed on 15 April 2025)	Orbits protocol
https://radiopaedia.org (Accessed on 15 April 2025)	Pineal and tectal plate protocol
https://radiopaedia.org (Accessed on 15 April 2025)	Pituitary gland protocol
https://radiopaedia.org (Accessed on 15 April 2025)	Posterior fossa protocol
https://radiopaedia.org (Accessed on 15 April 2025)	QUICK STROKE PROTOCOL
https://mrimaster.com/ (Accessed on 15 April 2025)	Soft Tissue Neck MRI/indication
https://radiopaedia.org (Accessed on 15 April 2025)	STROKE PROTOCOL
https://radiopaedia.org (Accessed on 15 April 2025)	Trigeminal neuralgia protocol
**Pediatric**	
https://radiopaedia.org (Accessed on 15 April 2025)	MR fetal indication
https://www.ohsu.edu/ (Accessed on 15 April 2025)	MR fetal protocol
https://www.ohsu.edu (Accessed on 15 April 2025)	MR Pediatric MSK
https://www.ohsu.edu (Accessed on 15 April 2025)	MR Pediatric Chest
https://www.ohsu.edu (Accessed on 15 April 2025)	MR Pediatric vascular malformation
https://www.ohsu.edu (Accessed on 15 April 2025)	MR Pediatric whole body
https://www.ohsu.edu (Accessed on 15 April 2025)	MR Peds abdomen
https://www.radiologyinfo.org (Accessed on 15 April 2025)	Pediatric MRI
**Spine**	
https://radiopaedia.org (Accessed on 15 April 2025)	Cervical spine protocol
https://radiopaedia.org (Accessed on 15 April 2025)	Lumbar spine protocol
https://radiopaedia.org (Accessed on 15 April 2025)	Thoracic spine protocol
**Ultrasound**	
https://geiselmed.dartmouth.edu/ (Accessed on 15 April 2025)	Ultrasound
**X ray**	
https://radiopaedia.org (Accessed on 15 April 2025)	Abdominal radiography
https://www.radiologyinfo.org (Accessed on 15 April 2025)	Chest Xray
https://radiopaedia.org (Accessed on 15 April 2025)	Cystography
https://geiselmed.dartmouth.edu/ (Accessed on 15 April 2025)	diagnostic protocol
https://radiopaedia.org (Accessed on 15 April 2025)	Hysterosalpingogram
https://www.radiologyinfo.org (Accessed on 15 April 2025)	Mammography
https://banffsportmed.ca (Accessed on 15 April 2025)	MSK
https://geiselmed.dartmouth.edu/ (Accessed on 15 April 2025)	orthopedic_MSK_protocols
https://imaging.heartofengland.nhs.uk (Accessed on 15 April 2025)	radiographic-standard-operating-protocols
http://rebalancemd.com (Accessed on 15 April 2025)	Standard-X-ray-Views
https://radiopaedia.org (Accessed on 15 April 2025)	Upper GI Xray
https://radiopaedia.org (Accessed on 15 April 2025)	Xray indications
